# Position-Controlled Data Acquisition Embedded System for Magnetic NDE of Bridge Stay Cables

**DOI:** 10.3390/s110100162

**Published:** 2010-12-24

**Authors:** Rocio Maldonado-Lopez, Rouven Christen

**Affiliations:** Empa, Swiss Federal Laboratories for Materials Science and Technology, Structural Engineering Research Laboratory, Ueberlandstrasse 129, CH-8600 Duebendorf, Switzerland

**Keywords:** data acquisition, embedded system, magnetic NDE, bridge stay cables

## Abstract

This work presents a custom-tailored sensing and data acquisition embedded system, designed to be integrated in a new magnetic NDE inspection device under development at Empa, a device intended for routine testing of large diameter bridge stay cables. The data acquisition (DAQ) system fulfills the speed and resolution requirements of the application and is able to continuously capture and store up to 2 GB of data at a sampling rate of 27 kS/s, with 12-bit resolution. This paper describes the DAQ system in detail, including both hardware and software implementation, as well as the key design challenges and the techniques employed to meet the specifications. Experimental results showing the performance of the system are also presented.

## 1. Introduction

Magnetic flux leakage (MFL) methods have been applied to the inspection of steel cables since the 1990s [1]. The working principle is that any discontinuity such as a broken wire or corrosion pitting distorts the magnetic flux in the sample under evaluation, and causes a local deflection of the magnetic fields lines at that point. Nowadays, it represents the most cost-effective and reliable inspection technique for non-destructive evaluation (NDE) of wire rope. A common application is the inspection of steel cables of aerial tramways, mining elevators and offshore pipelines [2–4].

In order to apply the same method on large scale structures such as stay cables of bridges, a number of additional design challenges have to be addressed. Due to the large diameter of stay cables, the generation of a magnetization level sufficient enough to obtain reliable data is a challenging task, requiring the development of new approaches in order to cope with this design problem.

These development activities lead to a MFL inspection device using electromagnets, which has been successfully applied in inspection of stay cables on bridges [5,6]. As a consequence, additional applications of magnetic NDE of large diameter cables, like automatic detection of flaws, based on artificial intelligence, or techniques for localization of flaws within the cross section of the cable [7–9] have been further developed.

In the present round of development, Empa is working in cooperation with a commercial partner, DMT Bochum, which has extensive know-how in the field of non-destructive and destructive tests on tension cables and their terminations. The goal of these joint efforts is the further development of the electromagnet device with the aim of increasing its efficiency and pursuing the commercialization of the method. A new design of the inspection device is being produced with the goal of delivering a measurement device suitable for routine testing of cables with a nominal diameter of more than 160 mm.

Within the framework of this collaboration, a custom-tailored sensing and data acquisition embedded system has been implemented, to be integrated in this new NDE device under development. The system has been designed to fulfill the requirements of the considered application in terms of data sampling rate, resolution and data storage capabilities, among others. Special attention has been paid to improve the efficiency and usability of the overall device, thus looking for the commercialization and industrial use of the device, in accordance to the main goals of the project. This paper focuses on the design of the sensing and data acquisition embedded system of the NDE inspection device. The main hardware and software design challenges and the implementation of the system will be described in detail. Experimental results showing the performance of the system will be presented and discussed.

## 2. System Architecture

### 2.1. System Requirements Analysis and Design Challenges

**Figure 1.**
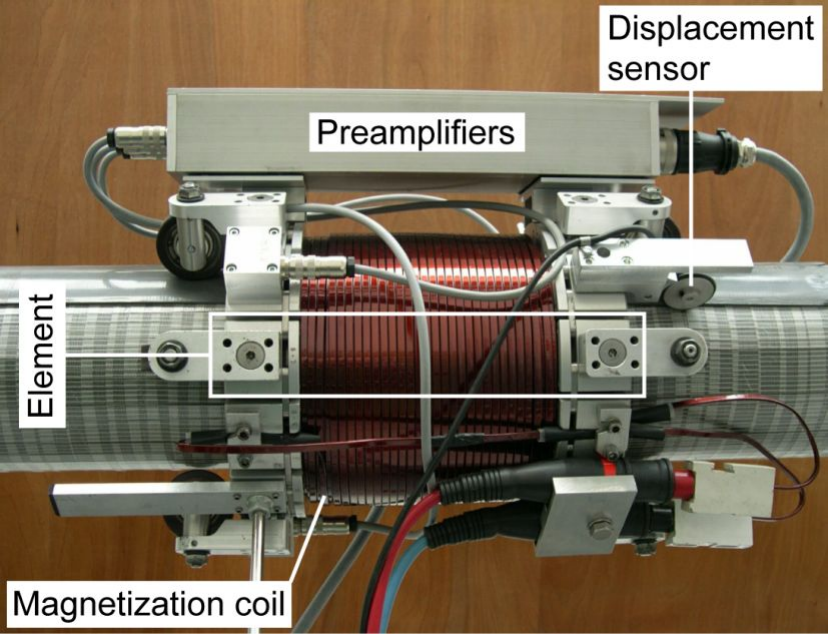
Inspection device developed at Empa [5,6].

The NDE inspection device presented in [5,6] is depicted in Figure 1, installed on a multi-strand cable. The mechanical part of this kind of devices is formed essentially by a coil or a configuration of coils responsible for the sufficient magnetization of the cable. The magnetic sensors that measure the magnetic flux on the surface of the cable (not visible in Figure 1) are integrated in the unit elements of the structure and arranged around the cable under inspection. The Data Acquisition system is embodied as well in the inspection device and its function is to read, convert and store the experimental data for further analysis. A displacement sensor included in the system permits a position-controlled data acquisition, while the inspection device is traveling along the cable.

This method is intended to be applied on large diameter cables with a length up to 400 m. Specifically, the diameter of such cables can range from *ϕ* > 160 mm to a maximum of *ϕ* < 250 mm. Once the NDE device is installed on the cable, the maximum distance between two magnetic sensors should not be more 15 mm. Therefore, considering a maximum diameter of *ϕ* < 250 mm and that sensors are equally distributed over the circumference, a maximum number of 50 magnetic sensors can be necessary. The DAQ system should be able to acquire data from this maximum number of data channels at the required sampling rate.

In the case of a DAQ system or data logger, the resolution and sampling rate are among the most important requirements. For the targeted application, a resolution of 12 bits is needed, and the minimal spatial resolution required is 1000Samples/m. Thus, one measurement from each sensor should be taken, for every 1mm displacement of the inspection device. Since the movement speed of the device is 0.5m/s, the system needs to be able to achieve a sampling rate of 500 Samples per second and channel. In terms of bit rate, for a 12-bit resolution, this means 8kbps per channel and 400kbps for all channels (considering 50 channels).

The maximum amount of data to be collected can be estimated as shown in Equation 1, where *L* is the maximum length of the cable, *f_S_* the sampling frequency, *Word_Size* is the number of bytes needed to store one single sensor read (2 bytes for a 12-bit resolution), and *N_Ch_* the maximum number of data channels.

(1) $$\mathrm{Data\_Size}=L\cdot f_{S}\cdot \mathrm{Word\_Size}\cdot N_{\mathrm{Ch}}=400\;m\;\cdot \;1000\;\;S/m\;\cdot \;2\;\mathrm{Bytes}\;\cdot \;50=40\;MB$$

The real-time data transmission to the base laptop, as deployed in other monitoring systems [10], is unreasonable due to the large amount of data and the required sampling rate. The adopted solution presented in this paper, relays on saving the data acquired during the inspection on a storage device, so that it can be accessed after the completion of the measurements. Nevertheless, the DAQ system is equipped with a low speed wireless serial link, which is used to control the inspection device.

Apart from the specifications already identified, concerning the data acquisition process, there are some other considerations related to the usability, effectiveness and commercialization feasibility of the system. One of the main goals is to come up with a portable NDE device, designed to simplify the assembly/disassembly procedures and to reduce the installation time. This requirement leads to strong constraints concerning the coil dimensions and weight, an aspect that is not covered in this paper, but also leads to the necessity of having a compact DAQ system. Therefore, the DAQ system has been integrated in a printed circuit board (PCB) and low-power components have been selected.

Another important aspect is the reliability of the acquisition system and the prevention of the loss of data. DMA (Direct Memory Access) modules are employed for speeding up data transfers. Data buffering and some recommendations from the SD card Association [11] have been implemented to enhance the writing process to the SD card. Error detection and control have been also implemented by software.

Finally, an important objective is to design the NDE system to be user friendly. The operator should be able to have control over the inspections, access the experimental data and analyze it without having a specific knowledge about how the data acquisition system internally works. Some design decisions were made to accomplish these goals:

- The inclusion of a Wireless link that provides a bi-directional communication channel with the DAQ system. A General User Interface (GUI) has been also implemented
- The storage of the experimental data in an organized manner onto an SD memory card, by the usage of a FAT file system
- The development of a data viewer tool that allows the user to conveniently visualize and analyze the acquired data

### 2.2. System Architecture Design

**Figure 2.**
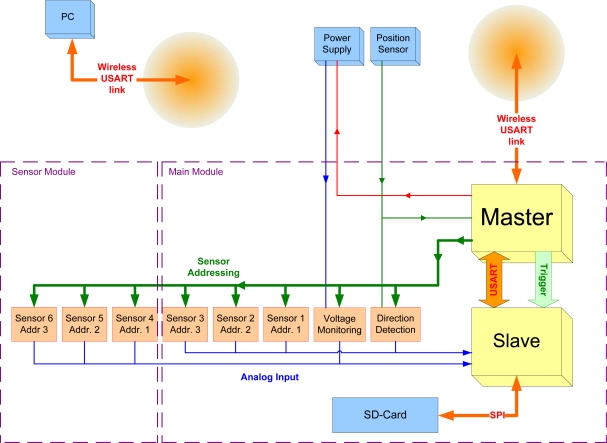
Sensing and data acquisition system in the inspection device.

A block diagram of the proposed DAQ system is shown in Figure 2. It consists of a main data acquisition PCB board as well as a number of individual sensor boards, organized in two different sensor arrays. Apart from the magnetic sensors, two additional data channels are present, related to the monitoring of the voltage in the coil and the direction of movement, respectively.

Among the tasks to be performed by the measurement system, some time-critical tasks can be identified. The generation of the control signals for the data acquisition needs to satisfy tight time constraints. The same applies for the management of the data buffering and the communication with the SD card. Due to the number of high demanding tasks to be performed concurrently, two micro-controllers (*μCs*) are used on the main DAQ board. Therefore, the work load can be shared between the two cores and the flexibility of the design is increased.

These two *μCs* are called *Master* and *Slave*, and communicate to each other through the Serial Port. The *Master μC* is in charge of the communication with the base laptop through the wireless link, as well as the generation of the triggering and addressing signals during the data acquisition. The *Slave μC* actually performs the data reading and conversion, and manages the communication with the SD card, using the SPI port. To take advantage of the multiple analog input channels available in the 12-bit ADC of the *Slave μC*, the data channels are separated into two arrays. That way, two sensors are selected at the same time, which improves the conversion performance.

The components of the Data Acquisition Board of the Main Module, as well as the software running onto the two *μCs* will be explained in more detail in Sections 3.1 and 3.3, respectively.

The Wireless link provides the user a way to have control over the conduct of inspections, by means of sending certain commands to the system. During the initialization phase, the operator can select the resolution and some other parameters related to the inspection to be performed, and send them to the system. A new file is created in the SD card to store the data to be acquired. Later, once the *Master μC* receives a *Start Inspection* command from the operator, the data acquisition process actually starts. To acquire the data at the right sampling rate, the *Master* generates the control signals taking into account the information from the position sensor. After every certain displacement of the inspection device, the *Master* selects all the data channels in the correspondent order and sets the triggering signal for the *Slave* to read the data from each selected channel. The *Slave* takes care about reading, converting and writing the data into the current data file in the SD card.

During the inspection, monitoring information, such as current speed of the inspection device, or possible errors are sent back to the operator. After the inspection has been completed, the operator can instruct the system to close the file and stop the data acquisition process.

## 3. System Functional Modules

### 3.1. Hardware Implementation: Main DAQ Board

**Figure 3.**
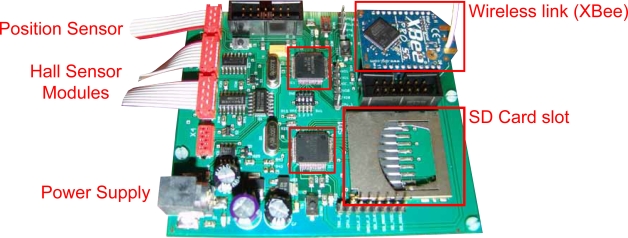
DAQ PCB Board.

A custom designed PCB board (Figure 3) containing the data acquisition hardware has been fabricated. The schematics and layout have been created using the freeware version of Eagle 5.6.0 PCB design tool [12]. Special attention has been given on minimizing the board dimensions (80 × 92 mm). The most important components present on the board are the two *μCs*, the XBee Wireless UART module, the socket for inserting the SD card, and the power supply circuit. Test points, buttons and LED’s have been added in order to simplify the debugging process of the hardware.

Regarding connectivity, apart from the Wireless link, two JTAG ports allow the programming of the *μCs*, whereas four additional connectors attach the board to the sensor arrays, the position sensor and the power supply of the coil, respectively.

The number of sensors to be read can be selected by hardware using a 4-way switch present on the DAQ board. Once the board is powered up, this number is read and stored by the *Master μC*.

Low-power *μCs* from the Texas Instruments MSP430 family have been selected for governing the DAQ board. This kind of devices are complete systems on-a-chip and include many integrated peripherals like converters, Direct Memory Access (DMA) modules, UARTs, *etc*. Moreover, they have a low power consumption and feature dedicate embedded emulation logic. All these characteristics make them a very attractive choice for this design.

From the specifications of the system, regarding speed and resolution (See section 2.1), the maximum acquisition time per channel should be 40 *μsecs* as calculated in Equation 2, where *f_s_* = 500 *Hz* is the sampling frequency derived from the movement speed of the device (*v* = 0.5 *m/s*) and *N* is the maximum number of channels.

(2) $$t_{\mathrm{sample}}\leq \frac{1}{f_{s}\;\cdot \;N}=\frac{1}{500\;\mathrm{Hz}\;\cdot \;50\;\mathrm{Channels}}\leq 40\mu \mathrm{secs}.$$

To achieve this requirement, the timing of the signals generated by the *Master* has been carefully designed. The time needed by software for addressing and reading one pair of sensors is 70 *μsecs*, which fulfills the specs. Thus, the Maximum Sampling Frequency that can be reached by the system can be expressed as:

(3) $$f_{\mathrm{sMAX}}=\frac{1}{70\;\mu \mathrm{secs}\;\cdot \;\frac{\mathrm{Channels}}{2}}$$

Moreover, the timing scheme implemented by the *Master* determines the time interval where the sensors remain selected and the data is available at the inputs of the analog to digital converter (ADC). Therefore, the sampling time of the ADC has to be set accordingly. There are some considerations regarding the minimum sampling time *t_sample_* required to obtain an accurate conversion, depending on the equivalent circuit at the analog input of the ADC. The analog input of the ADC can be modeled as a low-pass *RC* filter during the sampling time, which can have an influence on the accuracy of the conversion [13].

The minimum sampling time to achieve an accurate 12-bit conversion can be calculated as follows, where *R_S_* is the external source resistance, *R_I_* the internal input resistance, and *C_I_* the input capacitance of the MSP430 pin [13]:

(4) $$t_{\mathrm{sample}}\geq \left(R_{S}+R_{I}\right)\times \ln\left(2^{13}\right)\times C_{I}+800\;\mathrm{nsecs}.$$

The external source resistance *R_S_* depends on the interface circuit used to adapt signals coming from the sensors to the MSP430 levels. As an input interface circuit between the 3.6 V powered *μCs* and the 5 V sensor circuitry, a voltage divider circuit with an equivalent impedance *R_S_* of 10 *K*Ω has been employed. Thus, a minimum sampling time of 5.13 *μsecs* is required.

Taking the previous considerations into account, the sampling time has been set to a value of 16 *μsecs*. This corresponds to 128 cycles of the ADC clock, whereas the ADC clock frequency is 8 MHz.

### 3.2. Data Storage

The media chosen to store the experimental data acquired during an inspection is a Secure Digital memory card. Secure digital (SD) cards are widely used in portable devices due to their small size, relatively simplicity, low cost and low power consumption. SD cards up to 2GB formatted with FAT16 can be used with this system. The SD Card Association [11] has released a simplified version of the standard specifications for SD cards and SD hosts [14], describing the physical interface and the command protocol used by SD Memory Cards.

The MSP430 *Slave μC* employs a 3-bit SPI protocol to communicate with the SD card. This synchronous serial protocol is very popular for interfacing SD cards with *μCs* [15]. After writing a block to the SD card, the card needs some time for this data to be internally programmed (written). The SD card remains in a busy state and is not able to attend any other command during this time, which can be up to 150 ms [14]. As mentioned, data buffering is performed in the *Slave μC* to prevent the loss of data, but since the internal buffer capacity of the MSP430 is very limited, additional techniques have been implemented, to achieve a more efficient writing process. These strategies are described in Section 4 and experimental results showing their advantages are provided.

Moreover, the experimental data is stored in an organized manner inside the memory card, using a File Allocation Table (FAT16) file system. This way, the data can be easily accessed by the expert. Once the SD card has been connected to the host computer, the file system will be recognized by the operating system and the measurement files will be accessible.

The measurement files have a certain structure, being composed of a file header, a file body where the data is stored and a file tail. Before every inspection, the operator can use the GUI to set a name for the measurement file, in order to be able to identify each file afterwards. Additionally, parameters regarding the current inspection, like identification values, resolution, etc. can be also entered. This information is sent to the system and saved inside the file header, so that all the details related to the inspection are stored together with the experimental data in the same file. The next memory sector after the header is where the data area starts. The last sector in the file is used to store information about the lost sectors during the inspection (in case there are any). This information will be used by the file Viewer tool, to display the correct data corresponding to each position along the cable.

### 3.3. Firmware Implementation

The program running on the *Master μC*, manages the communication with the user and attends the user commands, like for example, starting or stopping an inspection. It also generates the data acquisition control signals. Furthermore, the *Data acquisition* program running on the *Slave μC*, performs the data conversion, data buffering and controls the communication with the SD card. The code has been written in C programming language, and has been developed and debugged using Eclipse IDE version 3.4.1, together with the Zylin Embedded CDT version 4.5.6 Plug-in for handling C projects optimized for embedded systems. As a C compiler, the MSPGCC, the GNU GCC tool-chain for the Texas Instruments MSP430 MCUs has been used.

In addition, various interface libraries have been created. In Table 1, a brief description of each library can be found. Some of them provide functions for communicating with the SD card using the SPI protocol, and store data using a FAT16 file system. Others provide common definitions regarding the communication protocols used.

The SD library requires the use of functions defined in the SPI library for controlling the communication with the SD card on a lower-level. Same way, some functions required by the FAT library are defined in the SD library. Thus, it can be considered that three communication layers are implemented, from a higher to a lower level, by the FAT, SD and SPI libraries, respectively.

The FAT library provides constants, data structures and functions, that allow to write data onto the SD card using a FAT file system. Due to memory limitations in the MSP430, only a reduced version of the FAT16 protocol has been implemented, including just the essential features needed for the targeted application. This way, the required RAM is maintained as low as possible. Only one partition and one directory are supported. It allows working with a single file at a time, and just writing operation to the files are supported. It is assumed that the memory device is as defragmented as possible, as the memory management capabilities are sacrificed in favor of optimizing the writing speed.

The communication protocol that allows the user to control the system, is based on a number of commands that can be sent from the user to the DAQ board, and error codes that are received back as answers to those commands (Table 2). Basically, four commands are supported, so the user can initialize the card, open a new file, or start and stop an inspection. The system performs some checks, like the correct connection of the card, memory availability, etc. Further the user is notified in the case of communication errors during the inspection, so that the expert can take a decision as soon as possible. Thanks to the error control performed by the system, possible malfunction can be detected, resulting in an optimization of the performance and required service time.

**Figure 4.**
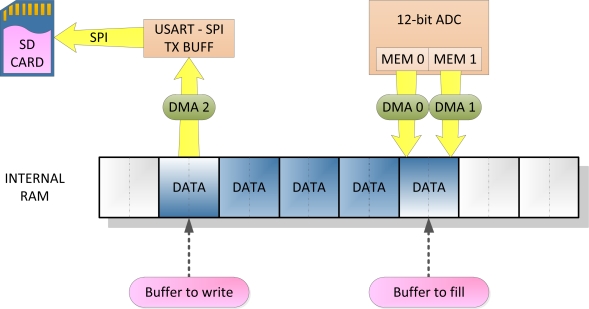
Modules involved in data writing. Example of internal data buffer.

Let us focus on the data acquisition strategy implemented in the *Slave μC*. A diagram showing the data structures and peripherals involved can be found in Figure 4. An array of memory buffers of 512 bytes each is used to temporarily store the acquired data while is being transferred to the SD card. Two pointers associated to the internal data buffers allow to control the incoming and outgoing data flows: *Buff_to_write*, that points to the buffer that is being sent to the SD card, and *Buff_to_fill*, that indicates the buffer that is being filled with new data from the sensors. The DMA (Direct memory access) capabilities available in the MSP430 *μC*, that permit fast data transfers without CPU intervention, are of a great advantage in applications where high sampling rates are required [16]. The strategy proposed in [16] also relies on the exploitation of the DMA capabilities.

As explained in Section 2.2, two of the input channels of the ADC are used. Thus, after the conversion procedure is completed, two new digital values are available in two internal registers of the ADC, MEM0 and MEM1. This event triggers the DMA channels 0 and 1, which transfers the digital values to the memory buffer pointed by *Buff_to_fill*. Every memory buffer in divided into 2 sub-buffers of 256 bytes. DMA0 links the MEM0 register of the ADC with the first sub-buffer, whereas DMA channel 1 connects the MEM1 register to the second sub-buffer. This way, the incoming data rate is optimized. Once a memory buffer is filled, an interrupt service routine (ISR) is executed and the pointer is redirected to the next free buffer in the array. In case that there are no more empty buffers available, the data in the last buffer is discarded and the buffer is reused, resulting in loss of data. The remaining DMA channel 2 transfers data from the buffer indicated by *Buff_to_write* to the UART1 transmission register, to be sent to the card. Once one buffer has been sent to the card, the DMA ISR updates the pointer, so the buffer gets free to be reused and get filled with new data.

**Figure 5.**
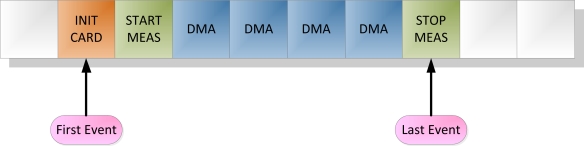
Example of event queue implemented in Slave micro-controller.

The performance of the program running on the *Slave μC* is based on attending the so-called *events* stored in the internal *event queue*. This data structure consists on a static array of elements, an example can be observed in Figure 5. Four different events are considered:

- INIT CARD: Initialization command from the user received.
- START MEAS: Start inspection command received.
- STOP MEAS: Stop inspection command received.
- DMA: A memory buffer has been filled with experimental data and is ready to be sent to the SD card.

Thus, every time a new command is received, or a new data buffer is ready to be sent to the SD card, the software generates the corresponding *event* and stores it in the *event queue*. The main function attends sequentially all the *events* in the *queue*. This way, no *event* that requires action from the Slave is left unattended. In case the program is busy and not able to attend an incoming *event*, it is stored to be attended later. For instance, at the end of a measurement, when the STOP command is received, the *event queue* structure allows sending every pending data buffer to the card, before the stop command gets attended and the measurement file gets closed. Same way, while the SD card is busy and is not accepting more blocks of data, some DMA *events*, as many as free buffers available in the internal array of buffers, can be entered in the *event queue* and are sent in a row to the card once it gets out of the busy state. Experimental results showing these capabilities will be presented in Section 4.2.

### 3.4. User Interface

Since the data acquisition is implemented on an embedded system, there is a need to create a graphical user interface (GUI) to allow for a comfortable way to control the NDE device and to accomplish the inspection task on site as efficiently as possible. Therefore, two different applications were implemented, written as Matlab© code using the GUI-Design-Environment (guide):

- A **Device Interface**, which allows the user to communicate with the NDE device, to acquire data and store it to the SD card as described in Section 3.3.
- A **Data Viewer**, which allows to open saved files from the SD card, in order to display and export the data.

**Figure 6.**
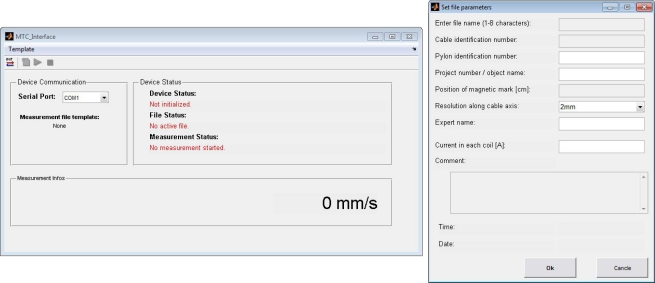
File header form in the device interface tool.

The device interface is a simple GUI, which is used to communicate with the *Master μC* of the DAQ board. There are only a limited number of commands available. Basically, the four push buttons in the toolbar correspond to the four possible actions: initialize device, create new file, start and stop measurement. The device interface takes care of the order the commands are sent to the controller. Those commands, which would not be accepted by the device due to its current state of operation, are disabled in the device interface software (e.g., before creating a file, the SD card has to be initialized). As described in Section 3.2, each file contains a header including information about the inspected object. This header information is entered using the device interface software. Every time the create new file command is executed, an input mask pops up as shown in Figure 6. The software also performs a validity check of the entered data (e.g., maximum number of characters, numbers or letters, lower/upper case).

Since some of the header information, such as project ID or expert name, are the same for a whole series of measurements, this information can be stored into template files.

**Figure 7.**
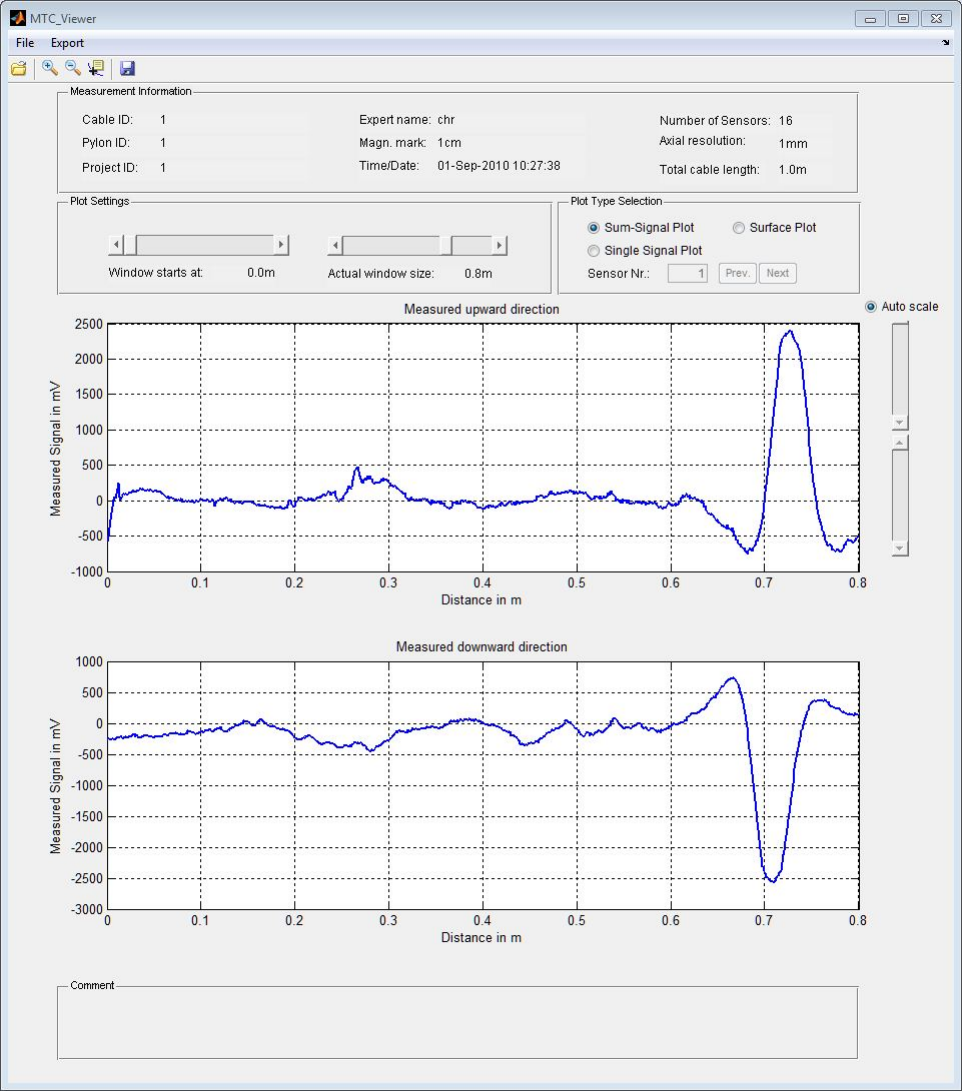
File header info displayed on data viewer tool together with the acquired data.

The data viewer can be used to read the measurement files stored on the SD card and display the data. This tool allows the expert to screen the captured data on site before the inspection device is removed from the cable. Once the data is classified as reasonable, the NDE device is dismantled and moved to the next cable. In order to navigate through the large amount of captured data, the display is divided into two screens, each showing one direction of measurement along the cable (see Figure 7). Since a signal caused by a flaw should be recognized in both measurements, this feature helps the expert to distinguish between wire breaks and interfering peaks in the signal. Using two sliders the starting point and the width of the displayed window can be changed, allowing the user to look through the data easily.

Further, the tool can be used for reporting purposes using its data export features. Because the data is stored in a binary format it is not directly accessible using Excel or a word processor, such as Notepad. The developed data viewer software reads the 16 bit words stored on the card and rearranges the data in order to get a *m* × *n* matrix, where *m* is the number of sensors and *n* is the number of measurements captured. This procedure is necessary, since the way the data is stored in the internal buffer of the *μC* is dictated by the usage of the DMA controller and its capabilities. Moreover, the software generates the displacement vector needed to display the data using the direction information (captured through the data channel attached to the position sensor) and the resolution, as shown in Equation 5.

(5) S(i)=S(i−1)+D(i)⋅ds

where, **S** is the reconstructed displacement vector, *ds* is the resolution in mm. and **D** is the measured direction information vector consisting of elements of +1 or −1, respectively.

As a further feature, two different export functions were implemented in the data viewer tool: an ASCII export and a graphical export of the actual view (screen-shot). Any ASCII export will certainly include all the information from the header, in order to assure, that the exported data can be assigned to the inspected cable.

## 4. Experimental Results and Discussion

### 4.1. Preliminary Tests

Several tests have been performed in order to debug the software and check the effectiveness of the strategies adopted to optimize the writing performance of the DAQ system, as well as its capability of overcome communication problems. The following strategies had been identified and adopted in order to minimize the risk of losing data blocks due to long busy periods after a writing operation on the SD card:

- The data block length should be the same as the sector size of the SD card, resulting in a more efficient data transmission
- Implementation of multiple block write mode instead of single block write mode
- Specification of the number of sectors to be written and pre-erase of the memory prior to the writing operation, so the internal data buffers of the SD card are used in a more efficient way
- The recommended size of the erase block *N_Blocks_* is dependent on the allocation unit size

**Figure 8.**
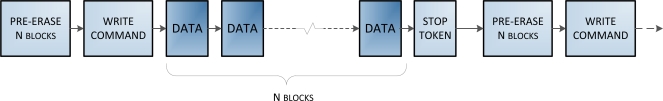
Multiple block write mode with pre-erase.

The writing process is depicted in Figure 8. Before every multi-block write operation, the number of blocks to be written *N_Blocks_* is specified and those blocks are pre-erased. After the write command is sent, *N_Blocks_* of data sectors are sent to the card in a row, followed by a stop token to indicate the end of the data transmission. A new writing cycle starts with a new pre-erase operation.

The software running on the *Slave μC* reads the internal standard registers of the SD card and extracts the parameters needed for calculating the optimum sector size and erase block size. This way, the optimization of the write procedure is independent of the SD card model in use.

The debugging test were carried out using a simple test set-up, prior to the installation of the DAQ system in the inspection device. A square wave signal from the signal generator acted as a trigger for the data acquisition, emulating the output signal from the position sensor. This way, the sampling frequency as well as the exact number of measurements to be taken could be controlled. During the initial tests 80 MB data were acquired at *f_s_* = 500 *Hz*, emulating a device movement speed of 0.5*m/s*.

As input data to be acquired by the system, a sine wave from the signal generator was applied to the analog inputs of the ADC. This way, every lost of data could be visually identified as signal jumps or discontinuities.

For comparison purposes, two different write modes were used: single block write mode and multiple block write mode. In some tests, the experimental data was recorded on the SD card from the very first sector available after the root directory, and in other cases the starting sector of the data area was aligned to an erase block boundary, as recommended in [14] for high-speed write operation on SD card. The size of the internal buffer was reduced to the minimum during these tests, only two memory blocks of 512 bytes each, to be able to compare the results in terms of lost sectors. These tests were performed using two different SD card models.

**Figure 9.**
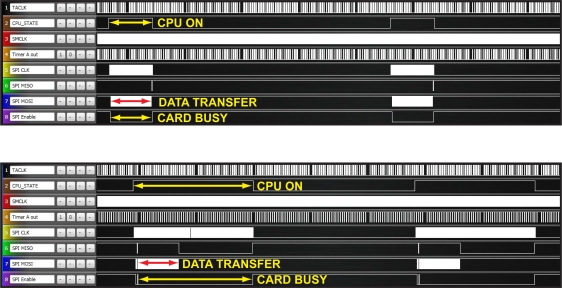
Single *vs*. multiple block write mode.

In Figure 9, the SPI interface signals from the scope are shown, when using single block write mode or multiple block write mode with pre-erase. Results show that, in the case of multiple block write mode, the CPU usage is more efficient, and the card remains busy less time for writing the same amount of data.

In the Table 3, results concerning the loss of data can be found. As can be noticed, both techniques, multiple block write mode and the alignment of data sectors with erase block boundaries, reduce the number of lost sectors significantly. Therefore, these two approaches have been adopted in the final version of the firmware. From these results, an estimation of the required buffer size was made, taking into account the number of sector lost and the sector size (512 bytes). It can be concluded that, at the required sampling frequency, the minimum data buffer size needed to avoid data loss is 5 kB. The F1611 derivative of the MSP430 used in the DAQ board provides 10kB internal RAM. Deducting the memory needed for storing variables, is possible to set the internal Data buffer size up to 9kB. Thus, up to 18 data blocks of 512 bytes can be stored, before start discarding data, in case the SD card stays busy.

### 4.2. Performance Tests

Additional performance tests have been carried out, acquiring data from two arrays of Hall sensors (16 data channels in total) at 1 kS/sec. The position sensor output was triggering the data acquisition. Signals coming from the hall sensors when being excited by a permanent magnet were measured.

**Figure 10.**
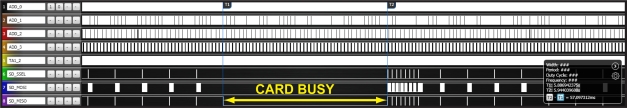
57 msec. delay with Kingston SD card.

During the performance tests, with a 9 kB internal data buffer, no data sectors were lost. The percentage of the internal data buffer used was also measured. It was found that 90% of the time only 2 out of 18 data sectors are used. In Figure 10, signals from the scope show a significant delay observed during these tests, while writing data on the SD card. In all cases, the data blocks acquired from the sensors, while the card remains busy, are stored in the internal data buffer of the *Slave μC*. It can be observed how these blocks are sent in a row as soon as the card is ready again.

## 5. Conclusions

A position-controlled, DMA-based data acquisition embedded system has been custom designed and fabricated, to be integrated in a novel inspection device for magnetic NDE of bridge stay cables under development at Empa, in the framework of the present collaboration between Empa and DMT Bochum.

The system provides a reliable data acquisition platform for the routine inspection of large scale cables, satisfying the requirements derived from the targeted application, in terms of sampling rate, resolution, data storage capabilities and power consumption.

Special attention has been paid on optimizing the usability of the system, with the aim of developing a device suitable for commercial use. A wireless communication channel is provided, which allows the expert to have control over the performance of the system, using an intuitive protocol. Techniques for facilitating the access and posterior analysis of the experimental data have been also implemented. Finally, error control procedures have been also included.

The performance of the system has been tested and experimental results are provided, showing the effectiveness of the strategies employed and the feasibility of the system for the use on magnetic NDE of bridge stay cables.

## Figures and Tables

**Table 1.** C libraries.

**fat.h**	Functions for implementing the File Allocation table (FAT16) file system
**sd.h**	Functions for high-level communication with the SD card
**spi.h**	Functions for controlling the SPI interface
**UART_comm.h**	Definitions for commands and error codes used in the high-level communication protocol between the GUI and the *μCs*, and between those and the SD card
**SDcomm_spi.h**	Definitions for low-level communication with the SD card
**states.h**	Definition of the states in the internal state machine of the main program and functions for management of the state machine
**events.h**	Definitions and function for managing the internal event queue in the *Data acquisition* program
**types.h**	Redefinition of data types

**Table 2.** Commands or events and error codes.

**Commands - Events**	**Error Codes**
Initialize SD card	Card Not Inserted
	Card Protected
	Card not initialized
	Communication Error

Open File	Existing filename
	Too many files in the card
	Card full
	Communication Error

Start Measurement	Write command not successful
	Communication Error

Stop Measurement	Communication Error

During measurement	Write command not successful
	Card full

**Table 3.** Data loss test.

	Single Write Mode		Multiple Block Write Mode	

	Data from first available Sector	Data aligned to Erase Block	Data from first available Sector	Data aligned to Erase Block
**Average number of lost sectors**	50 (25 kB)	12 (6 kB)	37 (18.5 kB)	8 (4 kB)
**Max. lost sectors in a row**	12	9	12	9
